# Risk factors of post-operative severe hyperlactatemia and lactic acidosis following laparoscopic resection for pheochromocytoma

**DOI:** 10.1038/s41598-017-00467-3

**Published:** 2017-03-24

**Authors:** Shubin Wu, Weiyun Chen, Le Shen, Li Xu, Afang Zhu, Yuguang Huang

**Affiliations:** Department of Anesthesiology, Peking Union Medical College Hospital, Chinese Academy of Medical Sciences and Peking Union Medical College, Beijing, 100730 P.R. China

## Abstract

Severe hyperlactatemia (SH)/lactic acidosis (LA) after laparoscopic resection of pheochromocytoma is an infrequently reported complication. The study aims to investigate the incidence of this complication and to determine the clinical risk factors. Patients who underwent laparoscopic resection for pheochromocytoma between 2011 and 2014 at Peking Union Medical College Hospital were enrolled. LA was defined as pH < 7.35, bicarbonate <20 mmol/L, and serum lactate ≥5 mmol/L; SH as lactate ≥5 mmol/L; and moderate hyperlactatemia (MH) as lactate 2.5–5.0 mmol/L without evidence of acidosis (pH > 7.35 and/or bicarbonate >20 mmol/L). Data concerning patient demographics, clinical history, and laboratory results were collected and statistical analyses were performed. Out of 145 patients, 59 (40.7%) developed post-operative hyperlactatemia. The incidences of MH and SH/LA were 25.5% and 15.2%, respectively. Multivariate analysis demonstrated that body mass index (BMI) (odds ratio [OR], 1.204; 95% confidence interval [CI], 1.016–1.426), 24-hour urine epinephrine concentration (OR, 1.012; 95% CI, 1.002–1.022), and tumor size (OR, 1.571; 95% CI, 1.102–2.240) were independent predictors of post-operative SH/LA. The data show that post-operative SH/LA is not a rare complication after pheochromocytoma resection and may be closely associated with higher BMI, larger tumor size, and higher levels of urine epinephrine.

## Introduction

Pheochromocytoma is a rare, catecholamine-producing neuroendocrine tumor originating from chromaffin cells of the adrenal medulla^1^. Cardinal manifestations of pheochromocytoma include episodic hypertension, headaches, sweating, and palpitations^1^. Although laparoscopic resection has become the first-line curative treatment for pheochromocytoma^2^, it is likely to induce wide fluctuations of circulating catecholamines intra-operatively. Excessive secretion of catecholamines may consequently augment glycolytic flux and decrease tissue perfusion, both of which have the capacity to boost lactate accumulation^3^.

Hyperlactatemia occurs when lactate generation exceeds lactate consumption. If not effectively controlled, moderate hyperlactatemia (MH) can progress to severe hyperlactatemia (SH) or even lactic acidosis (LA). LA, as a predictor of poor clinical outcome, may decrease myocardial contractility and cardiac output, can make the myocardium susceptible to cardiac arrhythmias, and can render the cardiovascular system insensitive to the effect of catecholamines^4^. In fact, previous studies have reported a myriad of cases of SH/LA in patients with pheochromocytoma^5–7^. However, owing to the rarity of pheochromocytoma, few studies have focused on the clinical factors resulting in post-operative SH/LA in patients who have undergone laparoscopic adrenalectomy.

The aim of this study was to evaluate the incidence of post-operative SH/LA and the clinical risk factors predisposing patients undergoing laparoscopic resection of pheochromocytoma to SH/LA.

## Results

Of a total of 145 patients, post-operative MH occurred in 37 and SH/LA in 22. The clinical and biochemical characteristics of patients with normolactatemia, MH, and SH/LA are shown in Tables 1–4. Pre-operatively, subjects in the group with SH/LA had larger tumors but a lower incidence of comorbid diabetes mellitus than those in the group with normolactatemia. However, there were no statistical differences in primary and secondary diabetes mellitus among the three groups. The SH/LA group had a greater proportion of epinephrine-secreting tumors than the normolactatemia group (Table 1). There were no statistical differences for pre-operative parameters among the three groups (Table 2). Intra-operatively, patients in the normolactatemia group experienced lower maximum heart rate and shorter anesthetic time than those in the MH groups, while the SH/LA group had a longer operative time than the normolactatemia group (Table 3). Post-operatively, subjects in the group with SH/LA had a longer mechanical ventilation time, greater maximum heart rate, and longer length of hospitalization than those in the normolactatemia group. The plasma lactate levels in the normolactatemia group were significantly lower than those of the MH and SH/LA groups (Table 4). In the multivariate analysis, body mass index (BMI), 24-hour urine epinephrine, and tumor size were independent predictors of post-operative SH/LA, with an odds ratio of 1.204 (95% confidence interval [CI], 1.016–1.426); 1.012 (95% CI, 1.002–1.022) and 1.571 (95% CI, 1.102–2.240), respectively (Table 5).

**Table 1:** Patient demographics and tumor characteristics.

Variable	Normolactatemia (*n* = 86)	MH (*n* = 37)	SH/LA (*n* = 22)	Overall *p* value
Age^a^ (years)	43.8 ± 14.9	43.5 ± 15.5	44.1 ± 13.2	0.992
Male sex, n (%)	36 (41.9)	20 (54.1)	9 (40.9)	0.424
BMI^a^ (kg/m^2^)	23.2 ± 3.1	23.2 ± 3.5	24.8 ± 3.0	0.096
ASA^b^	2.0 (2.0, 3.0)	2.0 (2.0, 3.0)	2.00 (2.0, 3.0)	0.582
Symptomatic, yes (%)	74 (86.0)	30 (81.1)	21 (95.5)	0.246
Genetic, yes (%)	11 (12.8)	5 (13.5)	2 (9.1)	0.863
Concomitant disease, n (%)				
Diabetes mellitus	18 (20.9)	7 (18.9)	0 (0)*	0.010
Primary	11 (12.8)	4 (10.8)	0 (0)	0.069
Secondary	7 (8.1)	3 (8.1)	0 (0)	0.181
Hepatic insufficiency	2 (2.3)	3 (8.1)	0 (0)	0.159
Coronary artery disease	3 (3.5)	1 (2.7)	2 (9.1)	0.511
Tumor size^a^ (cm)	4.3 ± 1.3	4.2 ± 1.5	5.3 ± 1.9*	0.015
Type of tumor, n (%)				
Epinephrine-secreting	13 (15.1)	6 (16.2)	9 (40.9)*	0.035
Norepinephrine-secreting	56 (65.1)	20 (54.1)	14 (63.6)	0.504
Dopamine-secreting	10 (11.6)	8 (21.6)	5 (22.7)	0.247
Tumor location, n (%)				
Left	38 (44.2)	15 (40.5)	7 (31.8)	0.571
Right	44 (51.2)	17 (45.9)	13 (59.1)	0.620
Bilateral	4 (4.7)	5 (13.5)	2 (9.1)	0.245

MH, moderate hyperlactatemia; SH/LA, severe hyperlactatemia/lactic acidosis; BMI, body mass index; ASA, American Society of Anesthesiologists’ classification.

*P < 0.05 vs the normolactatemia group. ^a^Mean ± standard deviation; ^b^median (25th percentile, 75th percentile).

**Table 2:** Pre-operative data.

Variable	Normolactatemia (*n* = 86)	MH (*n* = 37)	SH/LA (*n* = 22)	Overall *p* value
Pre-operative hemoglobin^a^ (g/L)	133.8 ± 17.4	136.7 ± 17.8	134.1 ± 10.7	0.662
Metformin, n (%)	9 (9.3)	3 (8.1)	0 (0)	0.148
Urine catecholamine^b^ (μg/day)				
Epinephrine	3.1 (2.1, 4.1)	2.6 (2.0, 4.5)	3.9 (2.5, 30.5)	0.063
Norepinephrine	119.8 (32.4, 315.6)	51.4 (28.3, 224.8)	69.1 (22.3, 142.6)	0.156
Dopamine	179.0 (123.4, 254.3)	177.4 (138.1, 317.1)	204.3 (137.3, 325.6)	0.423
Duration of *α* blockade^b^ (day)	31.0 (21.0, 40.3)	32.0 (20.0, 39.5)	31.0 (16.5, 41.0)	0.927
Dose of *α* blockade^a^ (mg)	28.2 ± 17.7	27.0 ± 16.0	30.3 ± 13.9	0.770
Number of antihypertensive medications^b^	1.0 (1.0, 1.0)	1.0 (1.0, 2.0)	1.00 (1.0, 2.0)	0.109
Pre-operative medications, n (%)				
*α* blockade	82 (95.3)	37 (100)	22 (100)	0.119
Selective *α* blockade	6 (7.0)	4 (10.8)	1 (4.5)	0.647
*β* blockade	13 (15.1)	9 (24.3)	7 (31.8)	0.175
Calcium channel blockade	5 (5.8)	2 (5.4)	4 (18.2)	0.192
Pre-operative MAP^a^ (mmHg)	101.7 ± 14.1	98.4 ± 15.2	101.3 ± 16.2	0.509
Pre-operative HR^a^ (bpm)	81.0 ± 14.8	80.8 ± 16.6	81.3 ± 11.4	0.994

MH, moderate hyperlactatemia; SH/LA, severe hyperlactatemia/lactic acidosis; MAP, mean arterial blood pressure; HR, heart rate.

*p < 0.05 vs the normolactatemia group. ^a^Mean ± standard deviation; ^b^median (25th percentile, 75th percentile).

**Table 3:** Intra-operative variables.

Variable	Normolactatemia (*n* = 86)	MH (*n* = 37)	SH/LA (*n* = 22)	Overall *p* value
Maximum SBP^a^ (mmHg)	172.4 ± 24.1	174.1 ± 21.7	179.3 ± 25.3	0.477
Minimum SBP^a^ (mmHg)	92.6 ± 10.0	92.2 ± 8.5	95.5 ± 9.7	0.392
Maximum HR^a^ (bpm)	98.9 ± 20.7	109.9 ± 12.9*	108.1 ± 10.1	0.003
Minimum HR^a^ (bpm)	60.5 ± 10.3	63.2 ± 12.5	62.3 ± 7.5	0.368
Propofol^a^ (mg)	158.5 ± 36.8	162.2 ± 41.7	162.3 ± 31.3	0.841
Total fluid intake^a^ (mL)	3296.5 ± 1041.0	3608.1 ± 1429.5	3809.1 ± 1491.9	0.148
Type of fluid infused, n (%)				
Crystalloid solution	86 (100)	37 (100)	22 (100)	—
Colloid solution	78 (90.7)	34 (91.9)	20 (90.9)	0.977
Intra-operative vasopressor, n (%)	68 (79.1)	28 (75.7)	17 (77.3)	0.915
Intra-operative vasodilator, n (%)	69 (80.2)	33 (89.2)	20 (95.2)	0.121
Epinephrine infusion, n (%)	2 (2.3)	1 (2.7)	2 (9.1)	0.386
Pneumoperitoneum^a^ (mmHg)	14.3 ± 0.5	14.3 ± 0.5	14.4 ± 0.7	0.790
Surgical approach, n (%)				
Transperitoneal	2 (2.3)	1 (2.7)	1 (4.5)	0.868
Retroperitoneal	84 (97.7)	36 (94.6)	21 (86.4)	0.135
Urine output^b^ (mL)	400 (300, 600)	500 (300, 800)	575 (300, 850)	0.306
Blood loss^b^ (mL)	100 (30, 300)	100 (50, 450)	100 (43, 300)	0.349
Anesthetic time^a^ (min)	161.2 ± 45.6	191.7 ± 69.6*	187.1 ± 51.0	0.007
Operative time^a^ (min)	109.5 ± 44.4	131.4 ± 67.7	140.2 ± 50.6*	0.016

MH, moderate hyperlactatemia; SH/LA, severe hyperlactatemia/lactic acidosis; SBP, systolic blood pressure; HR, heart rate.

*p < 0.05 vs the normolactatemia group. ^a^Mean ± standard deviation; ^b^median (25th percentile, 75th percentile).

**Table 4:** Post-operative parameters.

Variable	Normolactatemia (n = 86)	MH (n = 37)	SH/LA (n = 22)	Overall *p* value
Lactate^a^ (mmol/L)	1.6 ± 0.5	3.6 ± 0.7*	6.9 ± 2.1*	<0.001
Maximum SBP^a^ (mmHg)	159.9 ± 21.2	160.6 ± 16.0	168.0 ± 26.6	0.264
Minimum SBP^a^ (mmHg)	102.4 ± 15.5	103.2 ± 15.1	102.3 ± 17.0	0.969
Maximum HR^a^ (bpm)	100.4 ± 19.6	105.3 ± 16.2	110.8 ± 15.7*	0.045
Minimum HR^a^ (bpm)	66.2 ± 13.8	68.3 ± 16.3	70.3 ± 13.2	0.439
Mechanical ventilation^b^ (hour)	12.0 (5.5, 20.0)	11.0 (4.8, 21.5)	19.8 (7.5, 23.1)*	0.031
ICU stay^b^ (hour)	24.0 (21.5, 27.0)	23.0 (20.5, 27.8)	26.5 (23.5, 32.4)	0.053
Length of post-operative hospitalization^a^ (day)	4.8 ± 1.9	5.6 ± 2.7	6.1 ± 2.6*	0.028
Thirty-day mortality, n (%)	0 (0)	0 (0)	0 (0)	—

MH, moderate hyperlactatemia; SH/LA, severe hyperlactatemia/lactic acidosis; SBP, systolic blood pressure; HR, heart rate; ICU, intensive care unit.

*p < 0.05 vs the normolactatemia group. ^a^Mean ± standard deviation; ^b^median (25th percentile, 75th percentile).

**Table 5:** Uni- and multivariate analyses for predictors of post-operative SH/LA.

Variable	Univariate Analysis		Multivariate Analysis	
	*p* value	OR (95% CI)	*p* value	OR (95% CI)
BMI	0.034	1.171 (1.012–1.354)	0.032	1.204 (1.016–1.426)
Number of pre-operative medications	0.046	2.109 (1.014–4.390)	0.156	1.884 (0.786–4.518)
24-hour urine epinephrine	0.014	1.011 (1.002–1.020)	0.020	1.012 (1.002–1.022)
Operative time	0.065	1.007 (1.000–1.015)	0.499	1.003 (0.994–1.013)
Tumor size	0.006	1.567 (1.136–2.162)	0.013	1.571 (1.102–2.240)
Calcium channel blockade	0.054	3.683 (0.979–13.855)	0.235	2.668 (0.529–13.463)

BMI, body mass index; OR, odds ratio; CI, confidence interval.

## Discussion

Hyperlactatemia is a frequent complication of laparoscopic resection of pheochromocytoma (40.7%); SH/LA is also relatively common (15.2%). According to the results, patients with post-operative SH/LA are likely to experience a longer post-operative hospitalization, a higher post-operative maximum heart rate, and a longer duration of mechanical ventilation than those with normolactatemia. Our data demonstrate that patients with higher urine epinephrine levels, larger tumor size, and higher BMI are predisposed to post-operative SH/LA which potentially requires intensive care for management of elevated serum lactate.

Normally, there is a balance between lactate production and consumption. This can be reflected by the redox-coupled interconversion of pyruvate and lactate. Physiologically, the blood lactate-to-pyruvate ratio is 10:1, but it rises as the ratio of NADH concentration to NAD^+^ concentration (redox state) increases. Most pyruvate is generated by anaerobic glycolysis. Therefore, anaerobic glycolysis is the most common cause of hyperlactatemia or even lactic acidosis. However, aerobic glycolysis, stimulated glycolysis that depends on factors other than tissue hypoxia, also prompts lactate generation^8^. From the point of bioenergetics, generation of lactate ions by way of glycolysis is coupled with the production of an equivalent number of protons from the hydrolysis of the produced ATP^8^. In addition to glycolysis, the more efficient but slower ATP-producing process used by many cells is oxidative phosphorylation. In short, glucose plays a major role in lactate production, and any disorders correlated with hyperglycemia are likely to facilitate lactate production.

Catecholamines exert their effects via adrenoceptors in the majority of patients with pheochromocytoma^1^. In the human body, epinephrine and norepinephrine have overlapping but distinct effects on *α*- and *β*-adrenoceptors. At low doses, epinephrine acts predominantly on the peripheral *β*_1_- and *β*_2_- adrenoceptors. However, with increasing doses of epinephrine, the *α*_1_-adrenoceptor-mediated vasoconstrictor effect predominates. Norepinephrine acts predominantly on *α*-adrenoceptors to induce peripheral vasoconstriction, and has almost no effect on *β*-adrenoceptors. In addition, the metabolic effects of epinephrine are much stronger than those of norepinephrine^9^. According to the catecholamine effect, several mechanisms are adopted to explain the increase in blood lactate concentrations. The first is the metabolic effect of catecholamines. Not only do catecholamines have the ability to stimulate *α*_2_-adrenoceptors to inhibit insulin secretion^10^, they are also able to augment peripheral insulin resistance primarily through *β*-adrenoceptor agonism^11^. This phenomenon has been well documented in previous studies; insulin sensitivity improves after tumor resection in subjects with pheochromocytoma^12, 13^. Furthermore, catecholamines boost glycogenolysis and gluconeogenesis through *β*-adrenoceptor activation^14, 15^. Additionally, the stimulatory effect of epinephrine on glucagon secretion further augments these processes. The second is the vasoconstrictor effect of catecholamines, which may give rise to tissue anoxia, resulting in anaerobic metabolism and lactate overproduction in peripheral tissues.

In the present study, we found that urine epinephrine, but not norepinephrine, was a predictor of post-operative SH/LA in patients who had undergone laparoscopic adrenalectomy. This is consistent with previous studies. Administration of epinephrine has long been shown to result in a dose-dependent increase in lactate levels^16, 17^. In addition, previous clinical studies have demonstrated that extrinsic infusion of epinephrine induced elevation of plasma lactate levels, whereas extrinsic norepinephrine infusion had no effect^18^. The release of blood lactate was shown to be highest after tumor removal^5^. Furthermore, correlation of intra-operative lactate with urine epinephrine but not with norepinephrine has previously been demonstrated^5^. Hence, the metabolic effect of epinephrine may play a more important role in SH/LA after tumor resection. However, the exact mechanism behind the increase in lactic acid remains unclear in the present study. Indeed, lactate levels follow many more metabolic processes that are not related to tissue hypoxia. The lactate-to-pyruvate ratio might be helpful to distinguish the potential underlying mechanisms.

This study also suggests that tumor size is an independent predictor of post-operative SH/LA. Researchers have indicated that larger tumors possibly exert greater endocrine activity because tumor size is proportional to the urine catecholamine level^19, 20^. In this study, tumor size was correlated with 24-hour urine epinephrine level, consistent with previous studies. Furthermore, previous studies have shown that patients with larger tumors are not only more susceptible to hypertensive episodes intra-operatively due to massive catecholamine release, but are also likely to demand a higher dose of catecholamine infusion to maintain hemodynamic stability^19^. Therefore, tumor size, correlated with endocrine activity, has a substantial impact on lactate production.

Another finding of the study was that higher BMI independently predicted post-operative SH/LA. Other researchers have linked obesity to metabolic disorders^21^. Increased levels of circulating fatty acids, released by adipocytes in patients with obesity, impair muscular glucose uptake and utilization, leading to many of the metabolic changes observed^22^. Furthermore, tumor necrosis factor-*α*, generated by adipocytes, also impairs insulin action^23, 24^. In addition, a positive correlation was found between BMI and operative time, post-operative complications, and hospital stay in patients undergoing laparoscopic adrenalectomy^25^. The longer duration of operation may promote catecholamine release due to prolonged stimulation of the tumor. Thus, obesity, together with epinephrine, may facilitate lactate production by boosting glycometabolism.

Increased lactate levels usually reflect increased morbidity in patients undergoing cardiac surgery, in septic patients, or in patients after resuscitation^26, 27^. Furthermore, various studies have suggested that any increase in arterial lactate level is a strong and independent risk factor for death^28–30^. Thus, lactic acidosis has a high prognostic value. In the present study, patients with SH/LA had a longer duration of mechanical ventilation and post-operative hospitalization, reflecting more severe disease progression.

Unexpectedly, the incidence of diabetes mellitus in the SH/LA group was lower than that in the normolactatemia group. Diabetes mellitus is one of the metabolic complications of pheochromocytoma. Catecholamines are able to mobilise fuel in human body to meet the energy requirements, thus playing an vital role in carbohydrate metabolism. Twenty-five patients (17.2%) in the present study with pheochromocytoma concurrently had diabetes mellitus. However, the urine hormone levels in our study showed no difference between the three groups, which indicated that the incidence of diabetes mellitus secondary to pheochromocytoma was not statistically different between the three groups. Consequently, we divided diabetes mellitus into primary and secondary diabetes mellitus and that found neither made a difference among the three groups. Thus, secondary diabetes mellitus may be a confounder, impacting the result of Chi Square test.

The strength of this study is the high number of cases, given the low frequency of pheochromocytoma. There have been previous reports on the occurrence of SH/LA in this condition, but these are mainly case reports^6, 31^. Moreover, a multivariate analysis of risk factors has not been reported as yet. Although Suzuki *et al*. found that urine epinephrine level showed a moderate correlation with intra-operative peak plasma lactate level^5^, the logistic regression method used in that study did not meet the statistical conditions due to the limited sample size. Furthermore, besides urine epinephrine, the present study also found that BMI and tumor size were independent predictors of elevation of plasma lactate levels. This information is helpful for clinicians to evaluate and manage SH/LA more effectively.

The study presents several significant clinical findings. First, SH/LA was shown, for the first time, to be a relatively common complication after laparoscopic resection of pheochromocytoma; this warrants attention. Second, excessive catecholamine secretion as a result of pheochromocytoma resection may induce SH/LA, and the effect of epinephrine might be stronger than that of norepinephrine. Consequently, it is meaningful to monitor the real-time trends of blood lactate and catecholamine levels peri-operatively, especially for subjects with epinephrine-secreting tumors. Intensive care management of patients with elevated lactate may be required. Third, higher BMI and larger tumor size may exacerbate lactate generation. Thus, patients with two or three of the independent risk factors may be more susceptible to post-operative SH/LA. Clinicians should be aware of this in order to make appropriate preparations to avoid the complication.

Despite the limitations inherent in this single-center retrospective review, we found a relatively high incidence of post-operative SH/LA (15.2%). A multi-center prospective study may be desirable to further verify our conclusion. Unfortunately, the availability of peri-operative data, especially the post-operative lactate level, was limited because of the study’s design. Thus, it would be useful to monitor the post-operative lactate variation trend in a future study.

## Conclusions

In conclusion, post-operative SH/LA was a relatively frequent complication (15.2%) of laparoscopic resection for pheochromocytoma. A higher 24-hour urine epinephrine level, larger tumor size, and higher BMI were independent predictors of post-operative SH/LA.

## Methods

### Study population

We retrospectively enrolled 145 patients (>16 years old) who underwent laparoscopic resection for pheochromocytoma in our centre between March 2011 and June 2014. Those with an associated paraganglioma and those who experienced conversion were excluded from the analysis. Ethical approval (Ethical Committee No. S-K124) was provided by the Institutional Research and Ethics Committee of the Peking Union Medical College Hospital Beijing, China (Chairperson Prof Long-cheng Li). This committee waived the need for informed consent from all eligible patients.

### Laboratory and clinical parameters

In our centre, the blood lactate reference range was 0–2.5 mmol/L. MH was defined as serum lactate of 2.5–5.0 mmol/L without evidence of acidosis (pH > 7.35 and/or bicarbonate >20 mmol/L). LA was defined as pH < 7.35, bicarbonate <20 mmol/L and serum lactate ≥5 mmol/L. SH was defined as lactate ≥5 mmol/L. According to lactate level and acid-base status, patients were divided into three groups: the normolactatemia group, MH group, and SH/LA group. The first two groups were combined while performing univariate and multivariate analysis for predictors of post-operative SH/LA. Arterial blood samples were taken for measurement of lactate, performed using an arterial blood gas analyzer. Two blood samples were taken as soon as possible after the operation was finished. In our center, radial artery puncture is routinely performed before surgery to monitor intra- and post-operative invasive arterial blood pressure in patients with pheochromocytomata; this offers convenience for blood sampling. We used an arterial blood collection syringe (22 G × 1″BD Preset EclipseTM) produced by Becton, Dickinson and Company to take blood samples from the radial artery intra- and post-operatively. The post-anesthesia care unit is equipped with an ABL800 FLEX blood gas analyzer from Radiometer Medical. All blood samples underwent arterial blood analysis within 30–60 seconds of being obtained.

Variables comprised patient demographics, clinical history, laboratory data, and intra-operative details. Demographic data included age, sex, BMI, American Society of Anesthesiologists (ASA) classification, family history, clinical symptoms, and coexistent disease. Pre-operative laboratory analysis included serum hemoglobin and 24-hour urine catecholamine concentrations. Clinical data included duration and dose of alpha-blockade, kinds of pre-operative antihypertensive medications, tumor size, tumor location, length of intensive care unit stay, duration of mechanical ventilation, and length of post-operative hospitalization. Operative details included pre-operative mean arterial blood pressure measurement, pre-operative heart rate, maximum and minimum intra-operative systolic blood pressure measurements, maximum and minimum intra-operative heart rate, propofol dose, vasopressor and vasodilator usage, total volume of fluids administered, type of fluid infused, surgical approach, pneumoperitoneum pressure, volume of urine output, volume of blood loss, and anesthetic and operative durations.

### Statistical analysis

Continuous data were classified as being normally distributed or not by Q-Q plot. The mean ± standard deviation, median (25th percentile, 75th percentile), and n (%) were used to express normally distributed continuous data, non-normally distributed continuous data, and categorical data, respectively. All parameters were labelled in Tables 1–4 according to their data type. Overall differences between the three groups were evaluated using one-way ANOVA (normally distributed continuous data), Kruskal-Wallis test (non-normally distributed continuous data) and Chi square test (categorical data). For parameters with an overall *p* value < 0.05, *post-hoc* analysis followed the Bonferroni method (normally distributed continuous data) and the Nemenyi test (non-normally distributed continuous data), and partitions of the Chi square method (categorical variables) were performed for all pairwise multiple comparisons. Using multivariate logistic regression, we determined factors associated with post-operative SH/LA. And variables with a *p* value < 0.10 on univariate analysis were entered into a multivariate logistic regression analysis. Statistical significance was defined as *p* < 0.05. SPSS version 19.0 (IBM, SPSS, Inc.) was used to perform all statistical analyses.
